# Urokinase for thrombolysis in patients with acute ischemic stroke: rationale and design of a phase I dose-escalation study

**DOI:** 10.3389/fneur.2026.1750720

**Published:** 2026-04-15

**Authors:** Shengjun Wang, Qingfeng Ma, Xunming Ji, Wenbo Zhao, Longfei Wu

**Affiliations:** 1 Department of Neurology, Xuanwu Hospital, Capital Medical University, Beijing, China; 2 National Center for Neurological Disorders, Beijing, China; 3 Beijing Institute of Brain Disorders, Capital Medical University, Beijing, China

**Keywords:** acute ischemic stroke, dose-escalation, intravenous thrombolysis, maximum tolerated dose, urokinase

## Abstract

**Background:**

Previous evidence supports the efficacy and safety of intravenous urokinase thrombolysis in acute ischemic stroke (AIS), with additional advantages in terms of cost-effectiveness. Existing studies suggest that treatment response may be dose-dependent; however, current dosing practices remain largely empirical and lack standardization based on high-quality evidence. Establishing a weight-based dosing regimen is, therefore, essential to maximize therapeutic benefit and minimize risks.

**Objective:**

To determine the maximum tolerated dose (MTD) of urokinase and to develop an optimized weight-adjusted dosing protocol for AIS.

**Methods:**

OUTSET is a prospective, single-center, phase I, dose-escalation clinical trial using a weight-based, rolling-six design in patients with AIS. Patients who meet the inclusion criteria will be assigned to one of four dose groups (15,000 IU/kg, 20,000 IU/kg, 25,000 IU/kg and 30,000 IU/kg). Urokinase will be administered intravenously, diluted in 100 mL of normal saline, over a period of 30 min.

**Outcomes:**

The primary endpoint is the identification of the MTD based on dose-limiting toxicity events, including symptomatic intracranial hemorrhage, major bleeding, and other significant adverse events.

**Discussion:**

This study offers a structured, weight-based approach to determining the MTD of intravenous urokinase for AIS using a rolling-six dose-escalation design. By addressing the current lack of standardized, body-weight–adjusted dosing protocols, the findings may enhance the clinical safety and efficacy of urokinase thrombolysis. The results are expected to inform future phase II/III trials and support the development of individualized thrombolytic strategies in routine stroke care.

**Clinical trial registration:**

ClinicalTrials.gov identifier: NCT07047326.

## Introduction

Acute ischemic stroke (AIS) remains a leading cause of death and long-term disability worldwide. Intravenous thrombolysis (IVT) is among the most effective strategies for restoring cerebral perfusion and improving functional outcomes in AIS. However, the accessibility and utilization of IVT vary substantially across regions. While approximately 10–15% of patients in high-income countries receive IVT, this proportion falls below 2% in many low- and middle-income regions (1). Such disparities are influenced by multiple factors, including geographical and logistical barriers, yet economic affordability remains one of the most critical constraints (2). The high cost of recombinant tissue plasminogen activators such as alteplase and tenecteplase poses a major barrier to the widespread implementation of IVT, particularly in resource-limited settings (3–5).

Urokinase, a human-derived serine protease that directly converts plasminogen to plasmin, represents a cost-effective alternative thrombolytic agent (6). Decades of clinical experience and several clinical studies have demonstrated its efficacy in promoting recanalization and improving neurological outcomes in AIS. The Chinese Guidelines for the Diagnosis and Treatment of Ischemic Stroke recommend intravenous administration of 1.0–1.5 million international units (IU) of urokinase within 6 h of symptom onset (7, 8). In contemporary IVT practice, however, urokinase addresses a different research question from alteplase and tenecteplase. For alteplase, the weight-based regimen has already been well established (9, 10), whereas recent studies of tenecteplase have mainly focused on whether it may serve as a safe and effective alternative to alteplase in eligible patients with AIS (10–12). By contrast, for urokinase, the more immediate question is not simply whether it can replace another thrombolytic agent, but how its dosage should be optimized scientifically in current clinical practice. This issue is clinically important because urokinase remains relevant in China and other resource-limited settings owing to its affordability and accessibility, while available comparative evidence suggests that its effectiveness and safety may be broadly comparable to those of alteplase (6, 13, 14). Recent observational data further suggest that higher weight-adjusted doses (23,400–30,000 IU/kg) of urokinase may be associated with improved functional outcomes compared with lower-dose groups, without a corresponding increase in symptomatic intracranial hemorrhage (15). However, a weight-adjusted dosing regimen for urokinase thrombolysis remains unknown.

To address this critical knowledge gap, we designed the Dose-Escalation Safety Study of Urokinase for Thrombolysis in Patients with Acute Ischemic Stroke (OUTSET), a single-center, phase I clinical trial employing a rolling-six dose-escalation design. This study aims to determine the maximum tolerated dose (MTD) and to evaluate the safety and tolerability of weight-based urokinase thrombolysis, thereby establishing an evidence-based foundation for subsequent multicenter efficacy trials.

## Methods and analysis

**Figure 1:**
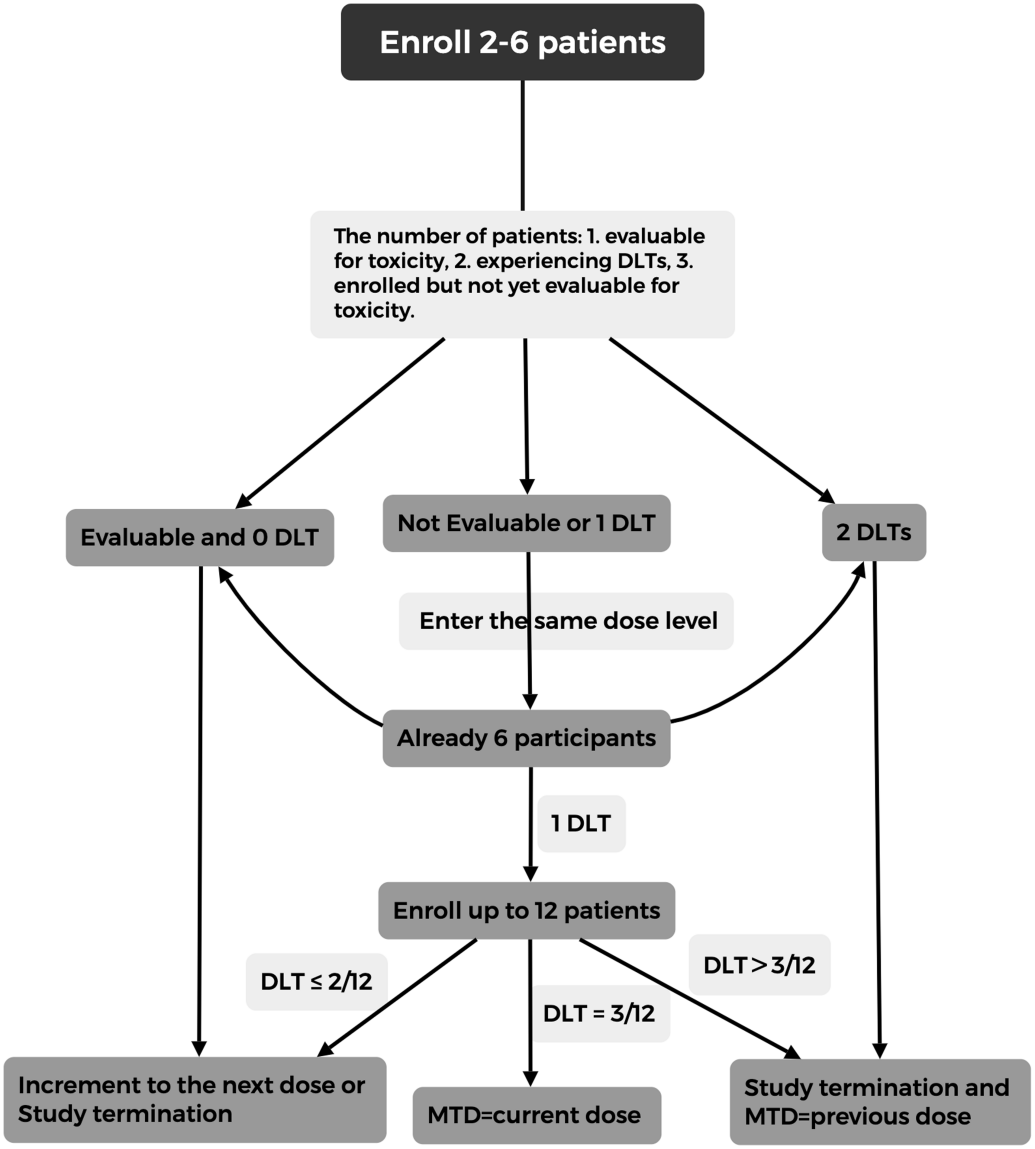
Rolling-six dose-escalation design for determining the MTD of intravenous urokinase in AIS. The figure shows the decision process based on the number of enrolled and evaluable patients, the occurrence of DLTs, and the number of patients still at risk for DLTs. Depending on these factors, dose escalation, cohort expansion, or termination decisions are made. The MTD is defined by the number of DLTs observed at each dose level. DLT, dose-limiting toxicity; MTD, maximum tolerated dose; AIS, acute ischemic stroke.

This study is a prospective, single-center, open-label, phase I, single-arm, dose-escalation clinical trial. A rolling-six design (16–19), a rule-based phase I dose-escalation method that allows concurrent enrollment of 2 to 6 participants at a given dose level, will be used (16). This design was selected because it reduces delays associated with sequential enrollment and can provide more safety information than the conventional 3 + 3 approach (16, 20). The study design is illustrated in Figure 1. The primary objective is to determine the MTD of urokinase thrombolysis in patients with AIS and to develop an optimal weight-based dosing regimen. This study has been approved by the Ethics Committee of Xuanwu Hospital and registered with Clinicaltrials.gov (Identifier: NCT07047326). All participants will provide written informed consent prior to enrollment in the study.

### Participants

Patients will be eligible for enrollment if they present with AIS. The inclusion criteria include:

1. Age 18–80 years.
2. AIS that can be treated within 6 h of symptom onset (the time of symptom onset is defined as “the last time point at which the patient appears normal”).
3. The National Institutes of Health Stroke Scale (NIHSS) ≥1, with symptoms considered clinically disabling or otherwise judged by the treating investigator to warrant intravenous thrombolysis.
4. Subjects or their guardians voluntarily sign the informed consent.

Exclusion criteria are as follows:

1. Head CT or MRI shows a large infarction (infarcted area >1/3 of the middle cerebral artery).
2. Unknown time of stroke onset.
3. Pre-stroke modified Rankin Scale (mRS) score ≥2.
4. NIHSS score 1A ≥2.
5. Intracranial hemorrhage (including parenchymal hemorrhage, intraventricular hemorrhage, subarachnoid hemorrhage, subdural/extradural hematoma, etc.).
6. A history of intracranial hemorrhage.
7. Propensity for acute bleeding, including platelet count <100 × 10^9^/L or otherwise.
8. Having received heparin treatment within 24 h.
9. On oral anticoagulants (e.g., warfarin) with INR >1.7 or PT >15 s;
10. Patients with planned or prior endovascular therapy.
11. A history of severe head trauma or stroke within 3 months.
12. Intracranial tumors, large intracranial aneurysms.
13. A history of intracranial or spinal surgery within 3 months.
14. A history of major surgery within 2 weeks.
15. Severe liver impairment (e.g., liver failure, cirrhosis, portal hypertension [esophageal varices], active hepatitis).
16. A history of gastrointestinal or urinary tract hemorrhage within 3 weeks.
17. Active visceral bleeding.
18. Aortic dissection found.
19. A history of arterial puncture at sites difficult for compression hemostasis within 1 week.
20. Life expectancy <1 year due to comorbid conditions.
21. Uncontrollable hypertension upon active antihypertensive treatment: systolic blood pressure ≥180 mm Hg, or diastolic blood pressure ≥100 mm Hg on ≥3 repeated measurements at 10-min intervals.
22. Blood glucose <2.8 mmol/L or >22.2 mmol/L.
23. Subjects who are unable or unwilling to cooperate due to hemiplegia after epileptic seizure or other neurological/psychiatric disorders.
24. Known to be allergic to urokinase.
25. Bacterial endocarditis, pericarditis, or acute pancreatitis.
26. Participation in other clinical trials within 30 days before screening.
27. Pregnancy, lactating women, or subjects who do not agree to use effective contraception during the trial.
28. Other conditions deemed by the investigator to impair adherence or pose risks to participants.

### Procedures

Eligible patients will be screened consecutively and enrolled at Xuanwu Hospital according to the predefined inclusion and exclusion criteria. Upon enrollment, patients will undergo baseline assessments, including electrocardiography, complete blood count, routine serum biochemistry (including liver function, renal function, electrolytes, and blood glucose), and coagulation studies (including prothrombin time, international normalized ratio, activated partial thromboplastin time, fibrinogen, and thrombin time). After informed consent is obtained, a baseline NIHSS examination will be performed (21), and patients will be treated with urokinase at the pre-specified dose calculated for their estimated body weight. In this study, the planned dose levels are 15,000 IU/kg, 20,000 IU/kg, 25,000 IU/kg and 30,000 IU/kg. No patient in any dosage tier will receive more than that dose calculated for a 100 kg person. These dose levels were selected based on the currently recommended empirical dosing range for intravenous urokinase and recent observational dose-exploration data. Dose escalation will proceed according to the following steps.

Firstly, 2 to 6 patients will be enrolled concurrently at the 15,000 IU/kg dose level. (a) If none of the evaluable patients at the current dose level experiences a dose-limiting toxicity (DLT, which is defined below), the dose will be escalated to 20,000 IU/kg. (b) If data for one or more enrolled participants is unavailable, or if one patient develops a DLT at 15,000 IU/kg, the new participant will enter at the same dose level. If one of the first 6 participants experiences a DLT, up to 6 additional participants (for a total of 12) will be enrolled at the same dose level to further assess safety and tolerability. If no more than 2 out of 12 patients experience DLTs, the dose will be escalated to 20,000 IU/kg. If exactly 3 out of 12 patients experience DLTs, the 15,000 IU/kg dose level will be considered the MTD. If more than 3 out of 12 patients develop DLTs, the previous dose (if applicable) will be defined as the MTD, and dose escalation will be halted. (c) If 2 patients experience DLTs at the current dose level before expansion, dose escalation will be terminated, and the previous dose level will be determined as the MTD.

The exact time of treatment initiation will be documented. Urokinase will be diluted in 100 mL of normal saline and administered via intravenous infusion over 30 min. Upon completion of thrombolysis, patients will be admitted to either the stroke unit or the neuro-intensive care unit and will subsequently receive secondary preventive therapy in accordance with established clinical guidelines (7). Fibrinogen levels will be measured at baseline and at 6 h post-treatment. Non-contrast computed tomography or magnetic resonance imaging will be performed within 36 h after treatment to detect hemorrhage, with additional imaging conducted in cases of clinical deterioration or at the discretion of the treating physicians. All imaging data will be independently reviewed for intracranial hemorrhage by two experienced physicians blinded to patient information. Discrepancies between reviewers will first be resolved by consensus; if disagreement persists, a third senior physician blinded to patient information will adjudicate the case. The final adjudicated result will be used for hemorrhage classification and related safety analyses. Neurological assessments using the NIHSS will be performed at baseline, 24 h, 36 h, and at 7 days or discharge after stroke onset. The Barthel Index (BI) and mRS will be assessed at 7 days or at discharge, and again at 3 months (22, 23). Ischemic stroke subtype will be classified according to the Trial of Org 10,172 in Acute Stroke Treatment criteria at 7 days or discharge, based on all available clinical diagnostic data (24). The mRS score at 90 days will be assessed either in person or via telephone interview. The schedule of enrolment, treatment, and follow-up assessments is summarized in Table 1.

**Table 1:** Schedule of enrolment, treatment, and outcome assessments in the OUTSET study.

Assessments	Screening/baseline	During urokinase infusion (0–30 min)	6 h post-treatment	24 h	36 h	Day 7 or at discharge	Day 90 (±7 days)
Eligibility assessment (inclusion/exclusion criteria)	×						
Written informed consent	×						
Demographic data	×						
Medical history and vascular risk factors	×						
Previous medication	×						
Physical examination and vital signs	×	×	×	×	×	×	
NIHSS	×			×	×	×	
Pre-stroke mRS	×						
Complete blood count	×						
Serum biochemistry	×						
Coagulation studies	×						
Fibrinogen	×		×				
Brain CT or MRI	×				×		
Urokinase administration		×					
Documentation of treatment start time		×					
DLT assessment		×	×	×	×		
Intracranial hemorrhage assessment					×		
Barthel index						×	×
Modified rankin scale						×	×
TOAST classification						×	
Adverse events and serious adverse events		×	×	×	×	×	×
All-cause mortality							×

NIHSS, National Institutes of Health Stroke Scale; mRS, modified Rankin Scale; DLT, dose-limiting toxicity; TOAST, Trial of Org 10,172 in Acute Stroke Treatment; CT, computed tomography; MRI, magnetic resonance imaging. Brain CT or MRI will be performed within 36 h after treatment to assess intracranial hemorrhage. Additional imaging may be performed in the event of clinical deterioration or when clinically indicated. Day 90 follow-up may be conducted during an outpatient visit or by telephone interview.

### DLT

We pre-defined the DLT before the start of the study, which included any of the following adverse events during the procedure:

- Symptomatic intracranial hemorrhage within 36 h after IVT (25).
- Parenchymal hemorrhage type 2 within 36 h after IVT (26).
- Major bleeding and clinically relevant non-major bleeding within 36 h after IVT (27, 28).

### Outcomes

The primary endpoint is the determination of the MTD based on DLTs. Secondary outcomes will be evaluated as follows: mRS score at 90 days; the proportion of patients achieving favorable functional outcomes, defined as an mRS score of 0–1, at 90 days; the proportion of patients exhibiting substantial neurological improvement on the NIHSS, defined as a decrease of at least 4 points, a score no more than 1, at 7 days or at discharge; fibrinogen level at 6 h post-treatment; symptomatic intracranial hemorrhage within 90 days, as defined by the European Cooperative Acute Stroke Study III (25); parenchymal hemorrhage type 2 within 90 days, as defined by the Safe Implementation of Thrombolysis in Stroke-Monitoring study (26); any intracranial hemorrhage within 90 days; major or clinically relevant non-major bleeding within 90 days (27, 28); BI score at 90 days; all-cause mortality within 90 days. Both serious and non-serious adverse events will be collected up to 90 days.

### Safety monitoring

An independent Data and Safety Monitoring Board (DSMB), composed of a neurologist, a neuroradiologist, and a methodologist who have no professional or financial relationship with the study investigators, is responsible for overseeing trial safety and progress. The DSMB reviews and evaluates all DLTs and adverse events on the basis of clinical symptoms, laboratory tests, and imaging data, providing independent recommendations to the investigators on whether the trial should be continued, modified, or terminated.

### Sample size

The sample size was determined by the rolling-six dose-escalation design rather than by a conventional efficacy-based power calculation. Under this design, 2 to 6 participants may be enrolled concurrently at each dose level, and dose-escalation decisions are based on the number of participants currently enrolled and evaluable, the number who experience DLTs, and the number who remain under DLT observation (16). Therefore, 6 participants represent the maximum initial evaluable cohort size for dose-escalation decisions at a given dose level, rather than the total number considered sufficient for the entire study (16, 20). Published applications of the rolling-six framework have shown that the total number enrolled at a given dose level may exceed 6 because non-evaluable participants can be replaced and additional patients may be enrolled to further characterize safety after a dose level has been judged acceptable (19, 20). In our protocol, participants who are not evaluable for dose-escalation decisions will be replaced according to the prespecified rolling-six rules, and if one DLT occurs among the first 6 evaluable participants at a dose level, that cohort may be expanded to 12 participants for further safety evaluation. With four planned dose levels, the expected total sample size ranges from 24 to 48 participants, depending on the occurrence of DLTs and cohort expansion.

### Statistical analysis

Given that this study employs a rolling-six design for dose escalation to identify an optimal weight-adjusted dosing regimen, the results will be summarized descriptively. Continuous variables will be presented as means (±standard deviation, SD) or medians (interquartile range, IQR), as appropriate, and compared using either the Student’s *t*-test or the Mann–Whitney *U* test. Categorical variables will be summarized as frequencies and percentages and compared using the Chi-square test or Fisher’s exact test. A two-sided significance level of 0.05 will be applied to all analyses. Statistical analyses will be performed using SPSS Statistics version 26 (IBM Corp., Armonk, NY, United States). All participants who receive urokinase will be included in the safety analysis. Reasons for withdrawal, dropout, or non-evaluable status will be recorded. Participants who are not evaluable for dose-escalation decisions will be handled according to the prespecified rolling-six rules.

## Discussion

Although urokinase has been widely used for thrombolysis in AIS for several decades in China, its optimal dosing strategy remains inadequately defined (13, 29–31). The currently recommended fixed-dose range of 1.0–1.5 million IU fails to account for individual variability in body weight and pharmacokinetics, leading to heterogeneous efficacy and safety outcomes in real-world practice, such variability may result in suboptimal treatment, including insufficient dosing that limits thrombolytic efficacy or excessive dosing that increases hemorrhagic risk. Establishing a scientifically grounded, weight-based dosing regimen is therefore essential to maximize thrombolytic benefit while minimizing the risk of ICH.

The study is designed to identify the MTD of urokinase in patients with AIS. The selected dose range of 15,000–30,000 IU/kg was based on current empirical clinical dosing practice and supported by recent observational dose-exploration findings suggesting that higher weight-adjusted doses (approximately 23,000–30,000 IU/kg) may be associated with better functional outcomes without an apparent increase in symptomatic intracranial hemorrhage (15). Accordingly, 15,000 IU/kg was selected as a conservative starting dose and 30,000 IU/kg as the upper boundary for dose escalation. The intermediate dose levels of 20,000 IU/kg and 25,000 IU/kg, using 5,000 IU/kg increments, were chosen to allow stepwise safety evaluation across the clinically relevant range while avoiding excessive dose gaps between adjacent cohorts. A prespecified cap at the dose calculated for a 100 kg person was further applied as an additional safety measure. Since observational evidence alone is insufficient to establish an optimized dosing strategy, this prospective dose-escalation study is needed to define the dose–toxicity relationship and identify the MTD, thereby informing subsequent phase II/III trials.

The rolling-six dose-escalation design allows cautious stepwise escalation guided by real-time safety data, with predefined DLT criteria to ensure participant safety. Continuous monitoring by an independent DSMB will further mitigate risk. We retained NIHSS ≥1 in the eligibility criteria because a low NIHSS score does not necessarily indicate a non-disabling stroke, and some patients with low scores may still have clinically meaningful deficits warranting intravenous thrombolysis. To reduce heterogeneity, however, enrollment was limited to patients with clinically disabling symptoms or those otherwise judged appropriate for intravenous thrombolysis, while patients with isolated mild, non-disabling deficits were excluded. Patients with planned or prior endovascular therapy were excluded to minimize confounding from mechanical reperfusion and to allow a clearer assessment of the dose–toxicity relationship of intravenous urokinase itself in this phase I setting (32, 33). Accordingly, the MTD identified in the present study should be interpreted as a foundational safety dose for patients treated without EVT, rather than as a dose directly generalizable to all patients with large-vessel occlusion. Future phase II/III studies should therefore evaluate the applicability of this dose in LVO populations separately, ideally through prespecified stratification, or, more preferably, a dedicated safety lead-in cohort in patients undergoing EVT. The study further incorporated an analysis of serum fibrinogen levels, aiming to offer an additional perspective for evaluating the risk of post-thrombolysis bleeding complications (34). By determining the MTD and characterizing the safety profile of weight-based urokinase thrombolysis, the OUTSET study will establish a scientific foundation for the next generation of clinical trials. The resulting evidence could support the development of standardized and affordable thrombolytic protocols, particularly in settings where urokinase remains a clinically relevant treatment option.

## Conclusion

The OUTSET study is designed to determine the MTD of intravenous urokinase using a weight-based, rolling-six design in patients with acute ischemic stroke. The findings will provide critical evidence to guide dose optimization and inform the design of future phase II/III trials aimed at establishing the efficacy and clinical utility of urokinase thrombolysis.
