# Supplementary material for: Urokinase for thrombolysis in patients with acute ischemic stroke: rationale and design of a phase I dose-escalation study
Source: Front Neurol. 2026 Apr 15;17:1750720. doi: 10.3389/fneur.2026.1750720 (PMC13124472; doi:10.3389/fneur.2026.1750720)
Supplement: Supplementary file 1 [file Table_1.DOCX]

Table 1. Schedule of enrolment, treatment, and outcome assessments in the OUTSET study

| **Assessments** | **Screening/Baseline** | **During urokinase infusion (0-30 min)** | **6 h post-treatment** | **24 h** | **36 h** | **Day 7 or at discharge** | **Day 90 (±7 days)** |
| --- | --- | --- | --- | --- | --- | --- | --- |
| Eligibility assessment (inclusion/exclusion criteria) | × |  |  |  |  |  |  |
| Written informed consent | × |  |  |  |  |  |  |
| Demographic data | × |  |  |  |  |  |  |
| Medical history and vascular risk factors | × |  |  |  |  |  |  |
| Previous medication | × |  |  |  |  |  |  |
| Physical examination and vital signs | × | × | × | × | × | × |  |
| NIHSS | × |  |  | × | × | × |  |
| Pre-stroke mRS | × |  |  |  |  |  |  |
| Complete blood count | × |  |  |  |  |  |  |
| Serum biochemistry | × |  |  |  |  |  |  |
| Coagulation studies | × |  |  |  |  |  |  |
| Fibrinogen | × |  | × |  |  |  |  |
| Brain CT or MRI | × |  |  |  | × |  |  |
| Urokinase administration |  | × |  |  |  |  |  |
| Documentation of treatment start time |  | × |  |  |  |  |  |
| DLT assessment |  | × | × | × | × |  |  |
| Intracranial hemorrhage assessment |  |  |  |  | × |  |  |
| Barthel Index |  |  |  |  |  | × | × |
| Modified Rankin Scale |  |  |  |  |  | × | × |
| TOAST classification |  |  |  |  |  | × |  |
| Adverse events and serious adverse events |  | × | × | × | × | × | × |
| All-cause mortality |  |  |  |  |  |  | × |

NIHSS, National Institutes of Health Stroke Scale; mRS, modified Rankin Scale; DLT, dose-limiting toxicity; TOAST, Trial of Org 10172 in Acute Stroke Treatment; CT, computed tomography; MRI, magnetic resonance imaging. Brain CT or MRI will be performed within 36 h after treatment to assess intracranial hemorrhage. Additional imaging may be performed in the event of clinical deterioration or when clinically indicated. Day 90 follow-up may be conducted during an outpatient visit or by telephone interview.
